# Comparison of late gadolinium enhancement patterns in different forms of cardiac amyloidosis

**DOI:** 10.1186/1532-429X-15-S1-P120

**Published:** 2013-01-30

**Authors:** Rebekka Kammerer, Katrin A Scherer, Fabian aus dem Siepen, Ralf Bauer, Stefan E Hardt, Sebastian Buss, Arnt V Kristen

**Affiliations:** 1 Cardiology, University of Heidelberg, Heidelberg, Germany

## Background

Cardiac involvement is common in different forms of amyloidosis and associated with limited survival. We aimed to use gadolinium (GAD) contrast-enhanced cardiac magnetic resonance imaging (CE-CMR) to characterize CE-CMR patterns that are related to the different forms of amyloidosis.

## Methods

In total, 115 patients (70 male, 45 female; mean age 60.7±1.2 years) with different forms of amyloidosis (AL n= 63, TTR n=52) were evaluated by evaluated by Vector-ECG gated 1.5T whole-body CMR (Achieva Intera^®^ Philips Medical Systems, Best, the Netherlands) and included SSFP and CE-CMR 2-,3-,4-chamber and short-axis planes. EDV, ESV, EF and myocardial mass were analyzed on a standard workstation. Longitudinal function was assessed by mitral (MAPSE) and tricuspid (TAPSE) annular plane systolic excursion. CE-CMR patterns were analyzed qualitatively (subepicardial, subendocardial, patchy) and semi-quantitatively (absent = 0, weak = 1, moderate = 2, severe = 3) in a modified 16 segment AHA model (maximal sum 48) of the left ventricle. Univariate and multivariate analysis were performed to define predictors of survival.

## Results

Patients with TTR amyloidosis were significantly older and had lower TAPSE (14.6±0.9 mm vs. 17.3±0.9 mm; p<0.05) as compared to patients with AL. Both cohorts did not differ in LV mass, LV ejection fraction, and MAPSE. CE-CMR was observed in 107 (93%) of the patients (AL n=59, 93.7%; TTR n=48, 92.3%). There was no difference between both forms of amyloidosis concerning sum of CE-CMR in the 16 segments by semi-quantitative analysis (AL 25.8±2.2; TTR 28.6±2.2).

In each of the 16 LV segments more severe CE-CMR was associated with an increase of LV mass as well as a decrease of LV ejection fraction, MAPSE and TAPSE, respectively (p<0.001). In TTR patients a more intensive CE-CMR was found in the subepicardial layer of the basal anterolateral (p<0.001), inferolateral (p<0.001) and of the midventricular inferolateral segment (p<0.001) as compared to AL patients.

During mean survival of 34.2±2.4 months there were 49 fatal events (death n=33; heart transplant n=16). Survival of AL patients was significantly worse as compared to TTR patients (p<0.05; logrank 6.225). In AL patients presence of subepicardial, subendocardial and patchy CE-CMR, LV mass, MAPSE, and TAPSE, but not LV ejection fraction, CE-CMR of LA, RA, RV or interatrial septum were predictors of survival by univariate analysis. By multivariate analysis subendocardial and patchy CE-CMR as well as LV mass were independent predictors of survival.

## Conclusions

Prevalence of CE-CMR is high and appears to be associated with the severity of cardiac dysfunction in different forms of cardiac amyloidosis. Survival of patients with AL amyloidosis was worse as compared to TTR amyloidosis. Besides LV mass subendocardial and patchy CE-CMR were independent predictors of survival in patients with AL amyloidosis.

## Funding

none

